# Safety and Efficacy of Tirofiban Combined With Mechanical Thrombectomy Depend on Ischemic Stroke Etiology

**DOI:** 10.3389/fneur.2019.01100

**Published:** 2019-10-29

**Authors:** Chao Sun, Xiang Li, Zheng Zhao, Xiangliang Chen, Chaoping Huang, Xuemei Li, Yajie Shan, Yang Zou, Yukai Liu, Mako Ibrahim, Linda Nyame, Baili Song, Fusang Wang, Xiaohan Zheng, Jue Hu, Zhihong Zhao, Junshan Zhou, Jianjun Zou

**Affiliations:** ^1^School of Basic Medicine and Clinical Pharmacy, China Pharmaceutical University, Nanjing, China; ^2^Department of Clinical Pharmacology, Nanjing First Hospital, Nanjing Medical University, Nanjing, China; ^3^Department of Neurology, Nanjing First Hospital, Nanjing Medical University, Nanjing, China; ^4^Department of Neurology, Changsha Central Hospital, Changsha, China; ^5^Department of Neurology, The First Affiliated Hospital (People's Hospital of Hunan Province), Hunan Normal University, Changsha, China; ^6^Faculty of Science, Melbourne University, Melbourne, VIC, Australia

**Keywords:** acute ischemic stroke, mechanical thrombectomy, tirofiban, safety, efficacy

## Abstract

**Background and Purpose:** The clinical use of tirofiban for patients with acute ischemic stroke (AIS) who underwent mechanical thrombectomy (MT) remains controversial. We aimed to evaluate the safety and efficacy of tirofiban combined with MT in AIS patients.

**Methods:** Patients with AIS who underwent MT from January 2014 to December 2018 were enrolled in three stroke units in China. Subgroup analyses were performed based on stroke etiology which was classified according to the Trial of ORG 10172 in Acute Stroke Treatment (TOAST) criteria. Safety outcomes were in-hospital intracerebral hemorrhage (ICH), symptomatic intracerebral hemorrhage (sICH) and mortality at 3-month. Efficacy outcomes were favorable functional outcome and functional independence at 3-month and neurological improvement at 24 h, 3 d and discharge.

**Results:** In patients with large artery atherosclerosis (LAA) stroke, multivariate analyses revealed that tirofiban significantly decreased the odds of in-hospital ICH (adjusted OR = 0.382, 95% CI 0.180–0.809) and tended to increase the odds of favorable functional outcome at 3-month (adjusted OR = 3.050, 95% CI 0.969–9.598). By contrast, in patients with cardioembolism (CE) stroke, tirofiban was not associated with higher odds of favorable functional outcome at 3-month (adjusted OR = 0.719, 95% CI 0.107–4.807), but significantly decreased the odds of neurological improvement at 24 h and 3d (adjusted OR = 0.185, 95% CI 0.047–0.726; adjusted OR = 0.268, 95% CI 0.087–0.825).

**Conclusions:** Tirofiban combined with MT appears to be safe and effective in LAA patients, but has no beneficial effect on CE patients.

## Introduction

Tirofiban, a short-acting non-peptide inhibitor of the glycoprotein (GP) IIb/IIIa receptor with high selectivity, can reversibly prevent platelet aggregation, and thrombi formation which play an important role in improving revascularization and clinical outcomes in acute ischemic stroke (AIS) patients (1, 2). Tirofiban combined with intravenous thrombolysis (IVT) was reported to be safe and effective in AIS patients (3–5). Recently, mechanical thrombectomy (MT) with second-generation devices has been recommended by AHA/ASA guidelines for AIS patients with intracranial large artery occlusion (6, 7). However, thrombectomy procedures can frequently lead to endothelial damage and subsequent platelets activation which may cause early reocclusion and clinical deterioration (8). In order to improve clinical outcomes, tirofiban has already been widely used in clinical practice in AIS patients treated with MT, even though the indication for AIS is not yet approved by US Food and Drug Administration. There were only four studies evaluated the feasibility of tirofiban combined with MT in AIS patients and results were not consistent (9–12). Whether the pharmacological advantage of tirofiban can evolve into satisfactory clinical outcomes remains under debate. Besides, previous trial which assessed clinical outcomes following IVT by stroke etiologies showed that when compared with patients with large artery atherosclerosis (LAA) stroke, those with cardioembolism (CE) stroke had worse clinical outcomes at 3-month follow-up (13). We assume such discrepancy also exists in stroke patients treated with MT. Based on this hypothesis, we conducted this study to examine the safety and efficacy of tirofiban combined with MT in LAA and CE patients.

## Methods

### Patients

This was a prospective study conducted in three stroke units in China (Nanjing First Hospital, People's Hospital of Hunan Province and Changsha Central Hospital). The study protocol was approved by the Ethics Committee at the local hospital. Written informed consent was obtained before enrollment. We analyzed clinical and radiological data for all consecutive patients with AIS who underwent MT from January 2014 to December 2018. Patients were considered eligible for MT if they met the following criteria: (1) had a primary diagnosis of AIS; (2) age ≥ 18 years; (3) onset of acute neurological symptoms <6 h; (4) intracranial large artery occlusion; (5) had a prestroke modified Rankin Scale (mRS) score of 0–2; (6) National Institutes of Health Stroke Scale (NIHSS) score ≥ 5 on admission. Exclusion criteria were: (1) history of intracranial hemorrhage before thrombectomy; (2) history of active bleeding or major surgery within 30 days; (3) platelet count <100 × 10^9^/L; (4) blood glucose concentration <2.8 mmol/L or >22.0 mmol/L; (5) severe hepatic or renal dysfunction. Arterial occlusion site was assessed by computed tomographic angiography (CTA), magnetic resonance angiography (MRA), or digital subtraction angiography (DSA). The stroke etiology were classified according to the Trial of ORG 10172 in Acute Stroke Treatment (TOAST) criteria: LAA, CE, and stroke of other determined or undetermined cause. Definitions of LAA and CE are shown in Supplementary File.

### Interventions

According to current guidelines for AIS, intravenous recombinant tissue plasminogen activator (rtPA) was administered in some of the enrolled patients evaluated within 4.5 h of symptom onset (6). The intravenous rtPA dosage was 0.9 mg/kg to a maximum of 90 mg. For those who had received intravenous rtPA therapy, cranial Computed Tomography (CT) imaging was conducted prior to MT to exclude hemorrhage transformation. After CT imaging confirming the absence of intracranial hemorrhage, MT procedures were performed immediately in the cooperation of the neurointerventionalist and stroke neurologist/physician. Direct MT without intravenous rtPA was performed in patients with heavy thrombus burdens (i.e., NIHSS score ≥ 20 on admission or thrombus length exceeds 8 mm). All patients treated with MT using a second-generation stent retriever device (i.e., Solitaire FR and Trevo). DSA was conducted to evaluate vascular condition after thrombectomy. After completion of MT, follow-up brain CT were performed immediately and 12–24 h later. The use of tirofban or not was at the judgment of neurointerventional specialists. Of the 195 enrolled patients, 71 were treated with tirofiban. Tirofiban was administrated intra-arterially if (1) the target artery remained occluded after thrombectomy (35 out of 71); (2) reocclusion of the recanalized artery (27 out of 71); (3) residual stenosis ≥ 50% in occlusion site after thrombectomy (5 out of 71); (4) multiple attempts with retriever during thrombectomy (≥ 3 times) (4 out of 71). Depending on the thrombus burden, a low-dose bolus of tirofiban was injected at a rate of 1 ml/minute (dose ranged from 0.25 to 0.5 mg). Bridging dual antiplatelet therapy (aspirin 100 mg and clopidogrel 75 mg) were given 4 h before the cease of tirofiban treatment. Patients in control group only received antiplatelet agent (aspirin 100 mg or clopidogrel 75 mg) after the completion of thrombectomy.

### Outcomes

The main safety endpoints were intracerebral hemorrhage (ICH), symptomatic intracerebral hemorrhage (sICH) and mortality at 3-month. ICH was assessed by CT or magnetic resonance imaging (MRI) scan. sICH was defined according to the ECASS III definition (14). The primary efficacy outcomes were favorable functional outcome and functional independence which were assessed by trained physicians employing mRS scores at 3 months with telephone questionnaires or clinic visits. A mRS score of 0–1 was considered as favorable functional outcome, a mRS score of 0 to 2 was considered as functional independence, and a mRS score of 6 indicated mortality. Secondary efficacy outcomes included neurological improvement at 24 h, 3 d and discharge. NIHSS scores were evaluated throughout the hospitalization to ascertain neurological improvement. Neurological improvement was defined as a reduction of at least 4 points on the NIHSS score.

### Statistical Analysis

All categorical variables are summarized as numbers and frequency (%), and chi-squared test or the Fisher exact test was conducted to detect differences between groups. For continuous variables, mean with standard deviation (SD) or median with interquartile range (IQR) are presented to summarize data, and between-group comparisons were performed via independent samples 2-tailed *t*-test or Mann-Whitney *U*-test. Multivariable logistic regression models were performed to further determine the association between tirofiban treatment and post-thrombectomy safety and efficacy outcomes. Results of regression analyses are expressed as odds ratios (ORs) with 95% confidence intervals (CIs). Variables that were considered as clinically relevant or with a potential association in the univariate analysis (*P* < 0.20) were included in the multivariate logistic regression analysis. Statistical analyses were executed by SPSS 22.0 (IBM Corp, Armonk, NY) with the significance level of *P* < 0.05.

## Results

### Baseline Characteristics

Table 1 displays the demographic and clinical characteristics according to tirofiban use. In this study, 195 AIS patients who underwent MT were recruited, including 71 receiving tirofiban as a combination therapy and 124 did not. In tirofiban group, the average age at onset was 66.2 ± 15.2 years old, 51 subjects (71.8%) were male, 24 (33.8%) were treated with intravenous rtPA. And the median baseline NIHSS score was 14 (IQR, 9-20), the median onset to groin puncture time was 340 (IQR, 215-505) minutes. Baseline characteristics were not significantly different between groups (*P* > 0.05 each). Demographic and clinical characteristics of subjects with LAA and CE are also summarized in the Table 1. Among these enrolled patients, 95 were diagnosed with LAA, 84 with CE. In each subgroup, the baseline characteristics were overall similar between tirofiban and non-tirofiban groups (*P* > 0.05 each).

**Table 1:** Patients' demographic and clinical characteristics.

**Baseline characteristics**		**All patients (*****n*** **=** **195)**			**Large artery atherosclerosis (*****n*** **=** **95)**			**Cardioembolism (*****n*** **=** **84)**		
		**Tirofiban (*n* = 71)**	**Without tirofiban (*n* = 124)**	***P* value**	**Tirofiban (*n* = 39)**	**Without tirofiban (*n* = 56)**	***P* value**	**Tirofiban (*n* = 28)**	**Without tirofiban (*n* = 56)**	***P* value**
Age, y		66.2 ± 15.2	66.3 ± 13.5	0.681	61.9 ± 10.0	66.0 ± 10.9	0.281	76.2 ± 11.5	68.5 ± 13.3	0.348
Males, *n* (%)		51 (71.8)	86 (69.4)	0.716	33 (84.6)	38 (67.9)	0.064	15 (53.6)	40 (71.4)	0.105
Smoking, *n* (%)		28 (39.4)	41 (33.1)	0.371	23 (59.0)	22 (39.3)	0.059	4 (14.3)	16 (28.6)	0.147
Diabetes mellitus, *n* (%)		16 (22.5)	23 (18.5)	0.503	11 (28.2)	9 (16.1)	0.154	5 (17.9)	12 (21.4)	0.701
Hypertension, *n* (%)		48 (67.6)	80 (64.5)	0.662	28 (71.8)	42 (75.0)	0.727	18 (64.3)	35 (62.5)	0.873
Hypercholesterolemia, *n* (%)		6 (8.5)	9 (7.3)	0.764	5 (12.8)	6 (10.7)	0.756	1 (3.6)	2 (3.6)	1.000
Atrial fibrillation, *n* (%)		21 (29.6)	45 (36.3)	0.340	0 (0)	2 (3.6)	0.511	21 (75.0)	39 (69.6)	0.608
Coronary heart disease, *n* (%)		15 (21.1)	27 (21.8)	0.916	3 (7.7)	8 (14.3)	0.516	12 (42.9)	18 (32.1)	0.334
Previous TIA/stroke, *n* (%)		2 (2.8)	0 (0)	0.131	0 (0)	0 (0)	–	2 (7.1)	0 (0)	0.108
Previous cerebral infarction, *n* (%)		10 (14.1)	24 (19.4)	0.351	5 (12.8)	11 (19.6)	0.382	4 (14.3)	11 (19.6)	0.546
Previous cerebral hemorrhage, *n* (%)		3 (4.2)	3 (2.4)	0.670	2 (5.1)	1 (1.8)	0.566	1 (3.6)	2 (3.6)	1.000
NIHSS on admission		14 (9–20)	15.5 (11–20)	0.559	11 (7–20)	14 (10–19)	0.204	19 (13–21)	16 (11–20)	0.198
Fasting blood glucose, mmol/L		6.54 (5.35–8.23)	6.30 (5.26–7.84)	0.383	6.13 (5.32–8.57)	6.29 (5.21–7.30)	0.615	6.75 (5.60–8.47)	5.86 (5.14–7.84)	0.243
Platelet, 10^9^/L		188 (157–233)	181 (143–220)	0.227	212 (170–248)	197 (162–234)	0.337	165 (115–189)	158 (138–203)	0.582
PT/INR		1.00 (0.93–1.10)	1.01 (0.94–1.12)	0.633	0.96 (0.92–1.03)	0.98 (0.90–1.06)	0.768	1.06 (0.99–1.14)	1.04 (0.98–1.16)	0.482
Intravenous thrombolysis, *n* (%)		24 (33.8)	55 (44.4)	0.149	13 (33.3)	23 (41.1)	0.444	9 (32.1)	27 (48.2)	0.161
Onset to groin puncture, min		340 (215–505)	301 (218–433)	0.697	357 (235–505)	355 (262–558)	0.401	307 (185–595)	256 (203–336)	0.281
IVT to groin puncture, min		71 (60–80)	69 (60–79)	0.472	72 (62–81)	67 (56–80)	0.414	68 (56–80)	72 (66–79)	0.251
Onset to recanalization, min		440 (290–602)	375 (305–540)	0.623	457 (360–600)	440 (342–688)	0.336	410 (282–670)	327 (266–419)	0.054
Permanent stenting, *n* (%)		7 (10.0)	6 (5.2)	0.211	6 (15.4)	4 (7.7)	0.246	1 (3.6)	2 (3.7)	0.976
Balloon angioplasty, *n* (%)		13 (18.6)	22 (19.0)	0.947	11 (28.2)	20 (38.5)	0.307	2 (7.1)	1 (1.9)	0.226
Anterior circulation stroke, *n* (%)		54 (76.1)	92 (74.2)	0.773	24 (61.5)	33 (58.9)	0.798	27 (96.4)	50 (89.3)	0.416
ICA		19 (26.8)	25 (20.2)	0.289	10 (25.6)	10 (17.9)	0.360	7 (25.0)	13 (23.2)	0.856
M1-MCA		20 (28.2)	39 (31.5)	0.631	7 (17.9)	17 (30.4)	0.171	12 (42.9)	17 (30.4)	0.256
M2-MCA		15 (21.1)	28 (22.6)	0.814	7 (17.9)	6 (10.7)	0.313	8 (28.6)	20 (35.7)	0.513
Posterior circulation stroke, *n* (%)		17 (23.9)	32 (25.8)	0.773	15 (38.5)	23 (41.1)	0.798	1 (3.6)	6 (10.7)	0.416
PCA		2 (2.8)	5 (4.0)	0.661	1 (2.6)	4 (7.1)	0.326	0 (0)	0 (0)	–
BA		10 (14.1)	21 (16.9)	0.600	9 (23.1)	16 (28.6)	0.550	1 (3.6)	3 (5.4)	0.717
VA		5 (7.0)	6 (4.8)	0.521	5 (12.8)	3 (5.4)	0.198	0 (0)	3 (5.4)	0.212
Reperfusion, *n* (%)		62 (87.3)	107 (86.3)	0.838	36 (92.3)	47 (83.9)	0.227	22 (78.6)	48 (85.7)	0.408
Stroke etiology	LAA, *n* (%)	39 (54.9)	56 (45.2)	0.345	–			–		
	CE, *n* (%)	28 (39.4)	56 (45.2)							
	Other, *n* (%)	4 (5.6)	12 (9.7)							

*TIA, indicates transient ischemic attack; NIHSS, National Institutes of Health Stroke Scale; PT/INR, prothrombin time and international normalized ratio; IVT, intravenous thrombolysis; ICA, internal carotid artery; M1-MCA, M1 segment of the middle cerebral artery; M2-MCA, M2 segment of the middle cerebral artery; PCA, posterior cerebral artery; BA, basilar artery; VA, vertebral artery; LAA, large artery atherosclerosis; CE, cardioembolism*.

### Intracerebral Hemorrhage

The results of intracerebral hemorrhage are summarized in Table 2. Overall, three patients (4.2%) treated with tirofiban experienced sICH and 14 patients (11.3%) not receiving tirofiban experienced sICH. No significant difference was detected in sICH between two groups (*P* = 0.092). The risk of in-hospital ICH was significantly lower in patients receiving tirofiban (18.3 vs. 33.9%; *P* = 0.020). In LAA and CE patients, the rates of sICH also were not significantly different between patients with and without tirofiban (*P* > 0.05 each). LAA patients treated with tirofiban were associated with lower risk of in-hospital ICH than patients in control group (10.3 vs. 32.1%; *P* = 0.013), however, such association was not observed in CE patients (32.1 vs. 33.9%; *P* = 0.870).

**Table 2:** Effects of Tirofiban treatment on intracerebral hemorrhage in patients with different ischemic stroke etiology.

	**Tirofiban**	**Without tirofiban**	***P* value**	**Adjusted OR (95% CI) and *P* value**
ALL PATIENTS^*^				
Sich	3/71 (4.2)	14/124 (11.3)	0.092	0.374 (0.102–1.374), 0.138
In-hospital ICH	13/71 (18.3)	42/124 (33.9)	0.020	0.382 (0.180–0.809), 0.012
**LARGE ARTERY ATHEROSCLEROSIS**^**#**^				
sICH	2/39 (5.1)	6/56 (10.7)	0.464	0.529 (0.093–3.000), 0.472
In-hospital ICH	4/39 (10.3)	18/56 (32.1)	0.013	0.280 (0.081–0.967), 0.044
**CARDIOEMBOLISM**^**$**^				
sICH	1/28 (3.6)	5/56 (8.9)	0.658	0.317 (0.029–3.482), 0.347
In-hospital ICH	9/28 (32.1)	19/56 (33.9)	0.870	0.692 (0.228–2.099), 0.516

* *Adjusted for age, gender, NIHSS on admission, previous TIA/stroke, intravenous thrombolysis*.

# *Adjusted for age, gender, NIHSS on admission, smoking, diabetes mellitus*.

$ *Adjusted for age, gender, NIHSS on admission, smoking, previous TIA/stroke, intravenous thrombolysis*.

*sICH, indicates symptomatic intracerebral hemorrhage; ICH, intracerebral hemorrhage*.

### Functional Outcome

**Figure 1:**
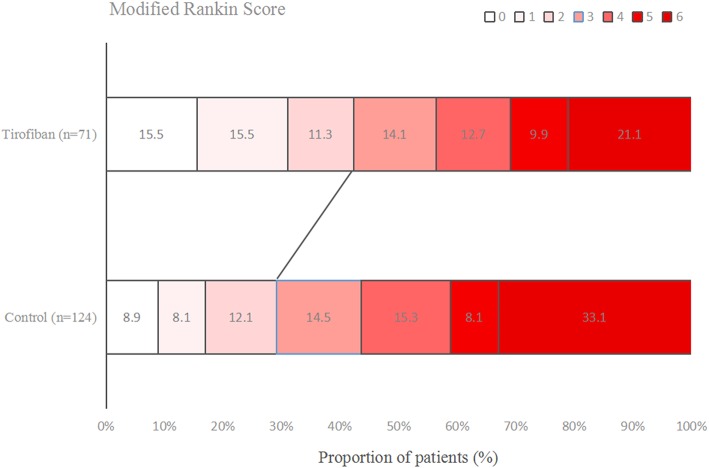
Distribution of mRS at 3-month in all stroke patients.

**Figure 2:**
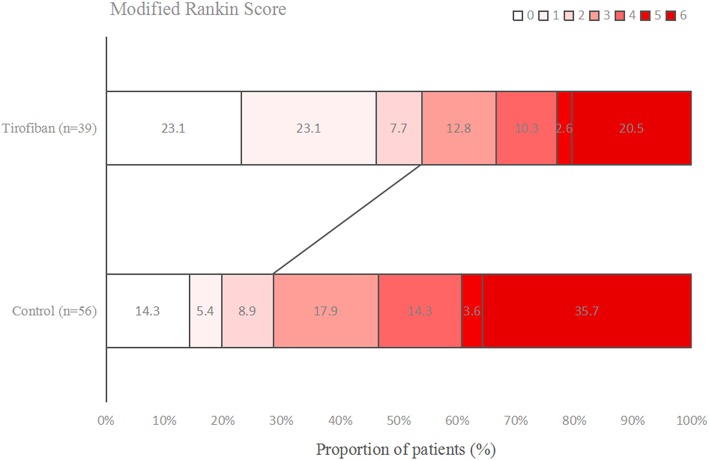
Distribution of mRS at 3-month in large artery atherosclerosis (LAA) stroke patients.

**Figure 3:**
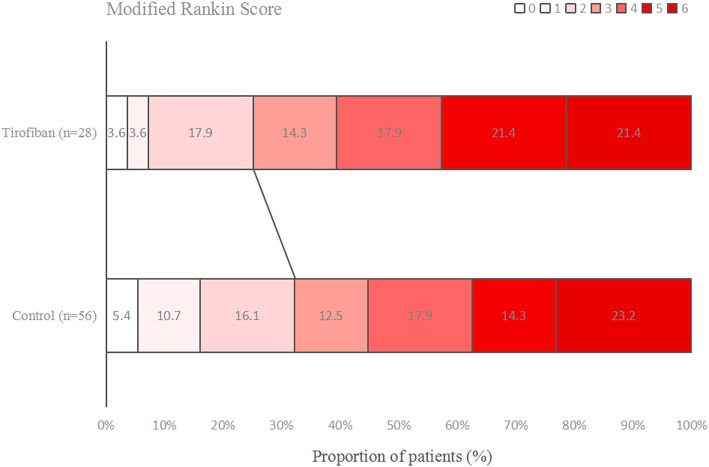
Distribution of mRS at 3-month in cardioembolism stroke patients.

Figures 1–3 present the distribution of mRS at 3-month in all stroke patients, LAA patients and CE patients, respectively. The results of functional outcome are presented in Table 3. At the 3-month follow-up, favorable functional outcome (mRS 0-1) occurred more frequently in tirofiban group than control group (31.0 vs. 16.9%; *P* = 0.023). However, tirofiban treatment was not associated with functional independence (mRS 0-2) and mortality (mRS = 6) (*P* = 0.060 and 0.076, respectively). In LAA patients, the incidences of achieving favorable functional outcome and functional independence were significantly higher in patients treated with tirofiban than those not receiving it (*P* = 0.006 and 0.013, respectively). In CE patients, all these outcomes mentioned above did not differ significantly between subjects with and without tirofiban (*P* > 0.05 each).

**Table 3:** Effects of Tirofiban treatment on functional outcomes in patients with different ischemic stroke etiology.

	**Tirofiban**	**Without tirofiban**	***P* value**	**Adjusted OR (95% CI) and *P* value**
ALL PATIENTS^*^				
3-mo mRS, 0-1	22/71 (31.0)	21/124 (16.9)	0.023	2.087 (0.902–4.827), 0.086
3-mo mRS, 0-2	30/71 (42.3)	36/124 (29.0)	0.060	1.862 (0.913–3.800), 0.087
3-mo mRS, 6	15/71 (21.1)	41/124 (33.1)	0.076	0.538 (0.256–1.133), 0.103
**LARGE ARTERY ATHEROSCLEROSIS**^**#**^				
3-mo mRS, 0-1	18/39 (46.2)	11/56 (19.6)	0.006	3.050 (0.969–9.598), 0.057
3-mo mRS, 0-2	21/39 (53.8)	16/56 (28.6)	0.013	2.281 (0.813–6.401), 0.117
3-mo mRS, 6	8/39 (20.5)	20/56 (35.7)	0.110	0.444 (0.145–1.359), 0.155
**CARDIOEMBOLISM**^**$**^				
3-mo mRS, 0-1	2/28 (7.1)	9/56 (16.1)	0.322	0.719 (0.107–4.807), 0.733
3-mo mRS, 0-2	7/28 (25.0)	18/56 (32.1)	0.500	0.945 (0.276–3.232), 0.928
3-mo mRS, 6	6/28 (21.4)	13/56 (23.2)	0.854	0.722 (0.202–2.577), 0.616

* *Adjusted for age, gender, NIHSS on admission, previous TIA/stroke, intravenous thrombolysis*.

# *Adjusted for age, gender, NIHSS on admission, smoking, diabetes mellitus*.

$ *Adjusted for age, gender, NIHSS on admission, smoking, previous TIA/stroke, intravenous thrombolysis*.

*mRS indicates modified Rankin Scale*.

### Treatment Effects

Details of treatment effects are provided in Table 4. The rates of neurological improvement at 24 h, 3 d and discharge were not significantly different between patients with and without tirofiban (22.5 vs. 29.8%, 28.2 vs. 37.1% and 59.2 vs. 47.6%, respectively, *P* > 0.05 each). In LAA patients, our results also proved that the rates of neurological improvement at 24 h, 3 d and discharge were not significantly different between two groups (*P* > 0.05 each). However, CE patients administrated with tirofiban had a significantly lower likelihood to have neurological improvement at 24 h and 3 d (*P* = 0.001 and 0.005, respectively).

**Table 4:** Effects of Tirofiban treatment on treatment effects in patients with different ischemic stroke etiology.

	**Tirofiban**	**Without tirofiban**	***P* value**	**Adjusted OR (95% CI) and *P* value**
ALL PATIENTS^*^				
Neurological improvement at 24 h	16/71 (22.5)	37/124 (29.8)	0.270	0.682 (0.342–1.364), 0.279
Neurological improvement at 3d	20/71 (28.2)	46/124 (37.1)	0.205	0.674 (0.354–1.283), 0.229
Neurological improvement at discharge	42/71 (59.2)	59/124 (47.6)	0.120	1.467 (0.802–2.684), 0.213
**LARGE ARTERY ATHEROSCLEROSIS**^**#**^				
Neurological improvement at 24 h	11/39 (28.2)	10/56 (17.9)	0.232	2.598 (0.850–7.942), 0.094
Neurological improvement at 3d	13/39 (33.3)	14/56 (25.0)	0.376	1.897 (0.696–5.172), 0.211
Neurological improvement at discharge	23/39 (59.0)	23/56 (41.1)	0.086	2.157 (0.869–5.358), 0.098
**CARDIOEMBOLISM^$^**				
Neurological improvement at 24 h	3/28 (10.7)	26/56 (46.4)	0.001	0.185 (0.047–0.726), 0.016
Neurological improvement at 3d	6/28 (21.4)	30/56 (53.6)	0.005	0.268 (0.087–0.825), 0.022
Neurological improvement at discharge	17/28 (60.7)	33/56 (58.9)	0.875	0.990 (0.334–2.931), 0.986

* *Adjusted for age, gender, NIHSS on admission, previous TIA/stroke, intravenous thrombolysis*.

# *Adjusted for age, gender, NIHSS on admission, smoking, diabetes mellitus*.

$ *Adjusted for age, gender, NIHSS on admission, smoking, previous TIA/stroke, intravenous thrombolysis*.

### Multivariate Regression Analysis

According to multivariate regression analysis, tirofiban was not increase the risk of sICH and mortality at 3-month (adjusted *P* > 0.05 each) nor was it associated with neurological improvement at 24 h, 3 d and discharge (adjusted *P* > 0.05 each). Whereas, we observed that tirofiban tended to improve the rates of favorable functional outcome and functional independence at 3-month (adjusted OR = 2.087, 95% CI 0.902–4.827; adjusted OR = 1.862, 95% CI 0.913–3.800). Intriguingly, a lower risk of in-hospital ICH was noted in tirofiban administrated patients (adjusted OR = 0.382, 95% CI 0.180–0.809). Multivariate regression analysis confirmed the safety of tirofiban in both LAA and CE patients (adjusted *P* > 0.05 each). In terms of efficacy outcomes, tirofiban exhibited strong potential to improve the odds of favorable outcomes at 3-month in LAA patients (adjusted OR = 3.050, 95% CI 0.969–9.598; adjusted OR = 2.281, 95% CI 0.813–6.401), while CE patients who were treated with tirofiban proved to have lower odds of achieving neurological improvement at 24 h and 3d (adjusted OR = 0.185, 95% CI 0.047–0.726; adjusted OR = 0.268, 95% CI 0.087–0.825).

## Discussion

High safety of tirofiban administration after MT have been demonstrated in our study. According to multivariate regression analysis, tirofiban was not associated with the risk of sICH and mortality at 3-month. Several previous studies also observed that endovascular treatment (EVT) alone has no absolute superiority over tirofiban combined with EVT in terms of safety (10–12, 15). Moreover, two recent studies that assessing the safety of abciximab (another approved GP IIb/IIIa inhibitors) combined with EVT concluded that abciximab treatment may be relatively safe (16, 17). Al-Mufti et al. found that abciximab administration after emergent carotid stenting caused no death at 3-month follow-up (16). And among all 99 patients treated with abciximab that Delgado et al. retrospectively assessed, no sICH was occurred (17). When compared with abciximab, tirofiban have a lower molecular weight and a shorter half-life, hence, tirofiban can penetrate into the thrombus easily and reduced platelet function can be normalized very shortly after the end of tirofiban administration (18). If abciximab after EVT was reported to be safe for AIS, then safety concern should not be the reason for withholding tirofiban therapy in EVT for AIS.

However, in the study by Kellert et al. they reported a higher risk of developing fatal ICH in patients receiving tirofiban (9). Several factors can account for this discrepancy. First, in this present study, tirofiban was administrated intra-arterially, while Kellert et al. treated patients with intravenous tirofiban. According to Kwon et al. intra-arterial use of tirofiban has several advantages compared with intravenous administration (19). One of the major superiority of intra-arterial tirofiban is due to the direct delivery of a concentrated dose to the target thrombus, effective thrombolysis can be achieved rapidly, and thus reduce possible bleeding events (19). Second, it is worth noting that patients in Kellert et al's study had higher NIHSS score on admission (9). Kellert et al. stated that median NIHSS score of patients received tirofiban was 18 (13, 35), however, median NIHSS score of patients received tirofiban in our study was 14 (9, 20). At last, 20 different catheters/thrombectomy devices/stent systems were used in the study by Kellert et al. some of which might lead to the excess risk for hemorrhagic complications (9).

Although tirofiban administration was not associated with neurological improvement, we still detected a strong tendency of tirofiban increasing functional independence rate in multivariate analysis, suggesting combined MT and tirofiban strategy is feasible and potentially efficacious when compared with MT alone. In the present study, functional independence at 3-month was achieved in 42.3% of patients. The reported rates of functional independence in previous RCTs of EVT ranged from 32.6 to 53.0% (20–24), which are comparable to our rate. Moreover, in 2017, Zhao et al. conducted a observational study attempted to investigate whether intra-arterial tirofiban was safe and effective in AIS patients undergoing MT with second-generation stent retrievers (10). They concluded that tirofiban seems to improve the odds of long-term functional independence which was consistent with our results (10). However, during the intervention, interventionists may be inclined to use tirofiban in patients with high possibility of having unfavorable outcome. As a result, this selection bias may have underestimated the efficacy of tirofiban.

Effects of tirofiban may depend on AIS etiology. In this study, we found that in patients with LAA stroke, tirofiban seems to lead to higher odds of neurological improvement at 24 h and favorable functional outcome at 3-month. By contrast, stroke patients of CE etiology who treated with tirofiban had significantly lower odds of neurological improvement (at 24 h and at 3d) and tended to have unfavorable outcomes at 3-month. Our findings may be attributed to the actual structure of thrombi. It is known that CE-induced occlusion are rich in red cells and are considered as red thrombi, whereas LAA-induced occlusion consist mainly of platelets which are referred to as white thrombi (13). Thus, tirofiban, as a non-peptide, short-acting GP IIb/IIIa antagonist which can prevent platelet aggregation, is beneficial to LAA patients by maintaining the reperfusion. Besides, in patients with occlusion related to intracranial atherosclerosis, tirofiban can stabilize the inflamed stenotic lesion and maintain blood flow which is helpful in preventing some ischemic events caused by inflammatory and platelet aggregation (25). Due to these aforementioned reasons, it may not be surprising that tirofiban is more effective in LAA patients. Moreover, atherosclerotic occlusion may be more difficult to achieve reperfusion with MT and reocclusion can frequently occur even after successful recanalization (8), so tirofiban as an adjuvant rescue strategy may be required. Therefore, tirofiban should be recommended to ischemic patients of LAA etiology, while tirofiban may not be suitable for CE patients because of the lack of efficacy. However, whether tirofiban treatment can adversely affect efficacy outcomes of CE patients treated with MT would be of interest as future research.

Certain limitations of our study need to be acknowledged. First, due to the relatively small number of patients treated with tirofiban enrolled in this observational study, high level evidence cannot be established. Further RCTs with a larger sample size are needed to have conclusive data for clinical practice. Second, the use of tirofiban or not was up to the neurointerventional specialists' discretion according to patient conditions during the procedure, which in turn leaves room for bias. Third, our study took an extraordinary effort to focus on patients with LAA and CE, as a result, other stroke etiologies of SVO, OD, UD as described in TOAST were overlooked. Last but not least, some parameters were missed. Perioperative managements may affect the risk of sICH and mRS scores at 3-month. However, these important baseline characteristics were not assessed in this present study. The influence of perioperative managements on post-thrombectomy outcomes may potentially exist.

## Conclusions

In LAA stroke patients, tirofiban combined with MT appears to be safe and potentially effective. By contrast, in CE stroke patients who underwent MT, tirofiban may not lead to better clinical outcomes. However, large RCTs are needed to further clarify our observation.

## Data Availability Statement

All datasets generated for this study are included in the manuscript/Supplementary Files.

## Ethics Statement

The studies involving human participants were reviewed and approved by The ethics committee of Nanjing First Hospital, Changsha Central Hospital and People's Hospital of Hunan Province. The patients/participants provided their written informed consent to participate in this study.

## Author Contributions

CS and XiL collected and analyzed the data, drafted and made critical revisions to the manuscript. ZheZ analyzed the data and performed all statistical analyses, and made critical revisions to the manuscript. XC, CH, XuL, YS, YZ, YL, MI, LN, BS, FW, XZ, JH, and ZhiZ treated the patients and collected the data, and made critical revisions to the manuscript. JZh and JZo conceived the study, treated the patients, collected and analyzed the data, drafted and made critical revisions to the manuscript.

### Conflict of Interest

The authors declare that the research was conducted in the absence of any commercial or financial relationships that could be construed as a potential conflict of interest.
