# Supplementary material for: Safety and Efficacy of Tirofiban Combined With Mechanical Thrombectomy Depend on Ischemic Stroke Etiology
Source: Front Neurol. 2019 Oct 29;10:1100. doi: 10.3389/fneur.2019.01100 (PMC6828979; doi:10.3389/fneur.2019.01100)
Supplement: Supplementary file 1 [file Data_Sheet_1.DOCX]

**Diagnosis of large artery atherosclerosis stroke and cardioembolism stroke**

Cardioembolism stroke was defined as the presence of atrial fibrillation, myocardial infarction in the past 6 months, or a high-risk source of embolism identified on echocardiogram according to Trial of Org 10172 in Acute Stroke Treatment criteria (1). Large artery atherosclerosis occlusion was diagnosed based on findings of prior MR angiography, postoperative digital subtraction angiography/MR angiography, or follow-up MR angiography >14 days from onset. All MRA images were read by two radiologists who were blinded to patients’ clinical information, and decisions were made by consensus. The degree of intracranial stenosis on MRA was calculated using the method of the WASID study (2) and was classified into four groups: <50% or no stenosis, 50% to 69%, 70% to 99%, and occlusion. The extracranial stenosis was evaluated with ultrasonography according to the published diagnostic criteria (3) or by contrast-enhanced MR angiography. In this present study, significant stenosis was defined as a more than 50% atherosclerotic stenosis or occlusion of the large intracranial and/or extracranial arteries. Based on the presence and location of cerebral atherosclerotic stenosis, patients were classified into three groups: 1) with only ICAS [69 out of 95]; 2) with only ECAS [7 out of 95] ; 3) with both ICAS and ECAS [19 out of 95] .

**References**

1. Albanese MA, Clarke WR, Adams HP Jr, Woolson RF. Ensuring reliability of outcome measures in multicenter clinical trials of treatments for acute ischemic stroke. The program developed for the Trial of Org 10172 in Acute Stroke Treatment (TOAST). *Stroke.* (1994) 25:1746-51. doi: 10.1161/01.str.25.9.1746

2. Samuels OB, Joseph GJ, Lynn MJ, Smith HA, Chimowitz MI. A standardized method for measuring intracranial arterial stenosis. *AJNR Am J Neuroradiol*. (2000) 21:643-6.

3. Grant EG, Benson CB, Moneta GL, Alexandrov AV, Baker JD, Bluth EI, et al. Carotid artery stenosis: grayscale and doppler ultrasound diagnosis-society of radiologists in ultrasound consensus conference. *Ultrasound Q*. (2003) 19:190-8.
